# Usefulness and reliability of cell free fetal DNA screening for main trisomies in case of atypical profile on first trimester maternal serum screening

**DOI:** 10.1186/s12967-019-02152-7

**Published:** 2019-11-28

**Authors:** Julie Carrara, Alexandre Vivanti, Jacques C. Jani, Adèle Demain, Jean-Marc Costa, Alexandra Benachi

**Affiliations:** 1 Service de Gynécologie-Obstétrique, Hôpital Antoine Béclère, AP-HP, 157 Rue de la Porte de Trivaux, 92140 Clamart, France; 2 Université Paris Saclay, 91190, Saint-Aubin, France; 3 Department of Obstetrics and Gynecology, University Hospital Brugmann, Université Libre de Bruxelles, Brussels, Belgium; 4 Laboratoire CERBA, Saint-Ouen l’Aumône, France

**Keywords:** Non-invasive prenatal testing, Cell free fetal DNA, Trisomy 21, Atypical maternal serum biomarkers, High HCG, Low PAPP-A, Intra uterine growth restriction

## Abstract

**Background:**

Patients with atypical values of HCG and/or PAPP-A are at higher risk of chromosomal abnormality and vascular complications of pregnancy. The performance of cfDNA in this particular population has not yet been evaluated.

**Objectives:**

The primary objective was to evaluate the usefulness and reliability of cfDNA in screening for trisomy 21, 18 and 13 for patients with HCG < 0.25 multiple of median (MoM), HCG > 5.0 MoM and/or PAPP-A < 0.25 MoM, PAPP-A > 2.5 MoM. The secondary objective was to evaluate the contribution of cfDNA assay for the prediction of pregnancy’s vascular complications.

**Method:**

Between June 2016 and July 2017, we analysed a women cohort from all over France who had at least one first trimester serum biomarker outside of normal range, in a retrospective, observational and multicentre study. Patients were included if they had a single pregnancy, normal first trimester ultrasound examination, whatever the result of the combined first trimester screening test was. The cfDNA was analysed by massive parallel sequencing technique. The accuracy of cfDNA assay was evaluated by calculation of sensitivity and specificity, and multivariate regression analysis was used to search for predictive factors for pregnancy’s vascular complications.

**Results:**

Among the 498 patients who underwent a cfDNA assay in this context, twenty-one (4.2%) were excluded because of loss to follow-up. Out of 477, test failure occurred for four patients initially, reduced to two patients (0.4%) after redrawn. CfDNA was positive for Trisomy 21 (n = 19), Trisomy 18 (n = 6) and Trisomy 13 (n = 1) and negative in 449. The sensitivity of cfDNA assay for trisomy 21 screening was 100% (19/19) (IC 95% 82.4–100) and specificity 100% (458/458) (IC 95% 99.2–100). Among the 447 patients included for prediction of vascular complications, there were four cases of pregnancy induced hypertension and 10 cases of preeclampsia, for which no predictive factor was identified. Intra Uterine growth restriction under 5th percentile (n = 44, 9.8%) was significantly associated with a low fetal fraction (OR = 0.87, IC 95% 0.79–0.96, p = 0.006).

**Conclusion:**

cfDNA assay is an effective and reliable tool for women with atypical profile of first trimester serum biomarkers.

## Introduction

Cell free fetal DNA (cfDNA) test has widely proved its performance in prenatal screening for trisomy 21, 18 and 13, in both high risk and general population [1, 2]. In the past few years, its use in clinical practice has been increasing constantly, due to its excellent sensitivity and specificity, added to its non-invasive character. Mainly because of its high cost, it is rarely used for now as a first-tier screening test, and it seems important to evaluate which patients benefit the most of this tool.

When results of first trimester serum biochemical markers [free β human chorionic gonadotropin (HCG) and pregnancy-associated plasma protein-A (PAPP-A)] are very different from the average value in general population, the software program calculating the risk cannot take into account the actual values, because they are inadequate to fit in the calculation equation [3]. Therefore, the software applies a truncation model, and uses the most extreme value useable but not the real one. This could lead to an underestimation of the calculated risk, and thus it has been described that these patients with abnormally high or low HCG or PAPP-A values have a higher risk of abnormal karyotypes [3]. These atypical values can contribute to placing the patient in the high risk group after the combined test, but not necessarily if the other criteria are in normal range (age and nuchal translucency at first trimester ultrasound). Thus, we have chosen to study women with first trimester screening test’s atypical profile (high or low HCG and PAPP-A rate), whatever the result of the combined test. In this population, it has also been described a higher risk of vascular complications during pregnancy, such as preeclampsia (PE) and intra uterine growth restriction (IUGR) [4–7]. In fact, several authors have suggested that there might be an association between those vascular risks and the fetal fraction of cfDNA [8–12]. It is known that this higher risk of vascular complications mostly concerns patients with high HCG and low PAPP-A, but as there is no data available concerning the link between cfDNA tests and those serum biochemistry atypical values, we chose to include also patients with low HCG and high PAPP-A in the analysis.

The main objective of this study was to evaluate the usefulness and reliability of cfDNA assay in screening for trisomy 21, 18 and 13 for patients with first trimester maternal serum screening’s atypical profile. The second objective was to evaluate the contribution of the cfDNA assay in the prediction of pregnancy’s vascular complications.

## Methods

### Study design

All cfDNA assays were performed in clinical setting. We conducted a retrospective, multicentric and nationwide study, including women from all over France who underwent a cfDNA assay sent to laboratory CERBA (as this laboratory is one of the few allowed to perform this test in France, and collaborates with medical healthcare practitioners from all over the territory) and who had at least one atypical biochemical marker of first trimester screening test. The inclusion criteria were women with an age of 18 or more, a singleton pregnancy, a normal first trimester ultrasound examination according to French national recommendation [13] (in particular nuchal translucency inferior to 95th percentile for gestational age), a first trimester screening test performed between 11 and 13.6 weeks of gestation (WG), a cfDNA assay, and at least one atypical value of first trimester serum assay (free ß HCG < 0.25 multiple of median (MoM), HCG > 5.0 MoM and/or PAPP-A < 0.25 MoM, PAPP-A > 2.5 MoM). The cutoff chosen for these values was decided in accordance with the most frequent and clinically relevant values found in literature [3]. Patients who did not do their first trimester ultrasound between 11 and 13.6 WG, as well as patients with second trimester screening test were not included. Patients with a vanishing twin described on early ultrasounds were not included, as it is well known that this could influence the level of HCG and PAPP-A assays, as well as the result of the cfDNA assay [14].

Pregnancy induced Hypertension (PIH) was defined as a blood pressure above 140 and/or 90 mmHg for women without any history of chronic high blood pressure. PE was defined as PIH associated with proteinuria above 0.3 g/24 h. Early PE was defined as PE with delivery before 37 weeks. Intra uterine growth restriction (IUGR) was defined as a birthweight lower than 10th percentile according to the Audipog growth curve [15]. It was considered severe if birthweight was lower than 5th percentile.

We collected all available computerized data from the selected patients and sent a survey to each health care practitioner who prescribed the cfDNA assay, either by regular mail or e-mail, in order to collect all pregnancy outcomes. In case of no-response or lack of information, the health care practitioners or the patients themselves were questioned by phone. All data were de-identified to ensure patient privacy and confidentiality. In line with French regulations regarding prenatal diagnosis, written informed consent was obtained from all patients. CERBA Laboratory is authorized by the Regional Health Agency to perform these screening tests. This research was approved by The Ethical Review Committee of the French CEROG (submission number CEROG 2018-OBST-0103).

### Cell free DNA analysis in maternal plasma

Maternal blood was collected in two cfDNA BCT Streck^®^ tubes (10 mL for each) and sent at + 4 °C to the clinical lab where plasma was isolated within 4 days after collection by a double centrifugation procedure and stored frozen at ≤ − 70 °C if not processed immediately. CfDNA in maternal plasma analysis was performed by massively parallel sequencing by using a whole genome approach, either by a home-brew assay as described previously [16] or by using the CE-IVD marked VeriSeq NIPT assay (Illumina^®^, Paris, France) since May 2017 due to the modification of the French regulation. During each run of the experiment, no template controls, plasma pooled from euploid pregnancies, or low positive controls prepared by mixing plasma from nonpregnant women and trisomy 13, 18, or 21 libraries were run simultaneously with the patient samples. Finally, sequence reads were mapped to the UCSC hg19 version of the human genome using Bowtie version 2; Z-scores were calculated for the targeted chromosomes 13, 18, and 21. The results are expressed as “positive” or “negative” when the experiments fulfilled the following metric criteria: library concentration 7.5 nM or greater, total number of aligned sequence reads nine million or greater and no amplification bias. Estimation of fetal DNA fraction from the plasma of pregnant women was performed either by using sequence read counts alone (referred to as SeqFF) [17] or in combination with fragment size distribution and Y chromosome-based method (VeriSeq NIPT).

### Statistical analysis

Descriptive results are reported as percentages for categorical variables and as median and range for quantitative variables. The performance of the test was characterized by specificity and positive predictive values. Exact 95% confidence intervals were computed with binomial distribution. Percentages were compared using Chi squared test or Fisher’s exact test. Mann–Whitney U test was used to compare medians.

Univariate analysis was used to investigate if vascular pregnancy complications were associated with different factors, such as method of conception (in vitro fertilization or natural conception), ethnicity, multiparity as categorical variables and maternal age (years), weight (kg), gestational age at test (weeks of gestation), HCG and PAPP-A levels, fetal fraction of cell free DNA as continuous numerical variables. Multiple logistic regression analysis was subsequently performed to determine the significant independent contribution of those variables yielding a p < 0.1 in the univariate analysis. A subgroup analysis was made to determine if a particular group of patients was at risk of vascular complications (group with elevated level for both PAPP-A and HCG, group with low level for both biomarkers, or mixed).

Data were analyzed with the statistical software MedCalc, version 15.11.4 (Mariarkerke^®^, Belgium) and Excel version 15.0 (Microsoft^®^, Redmond, Wash). A two-sided p-value of less than 0.05 was considered statistically significant.

## Results

**Fig. 1:**
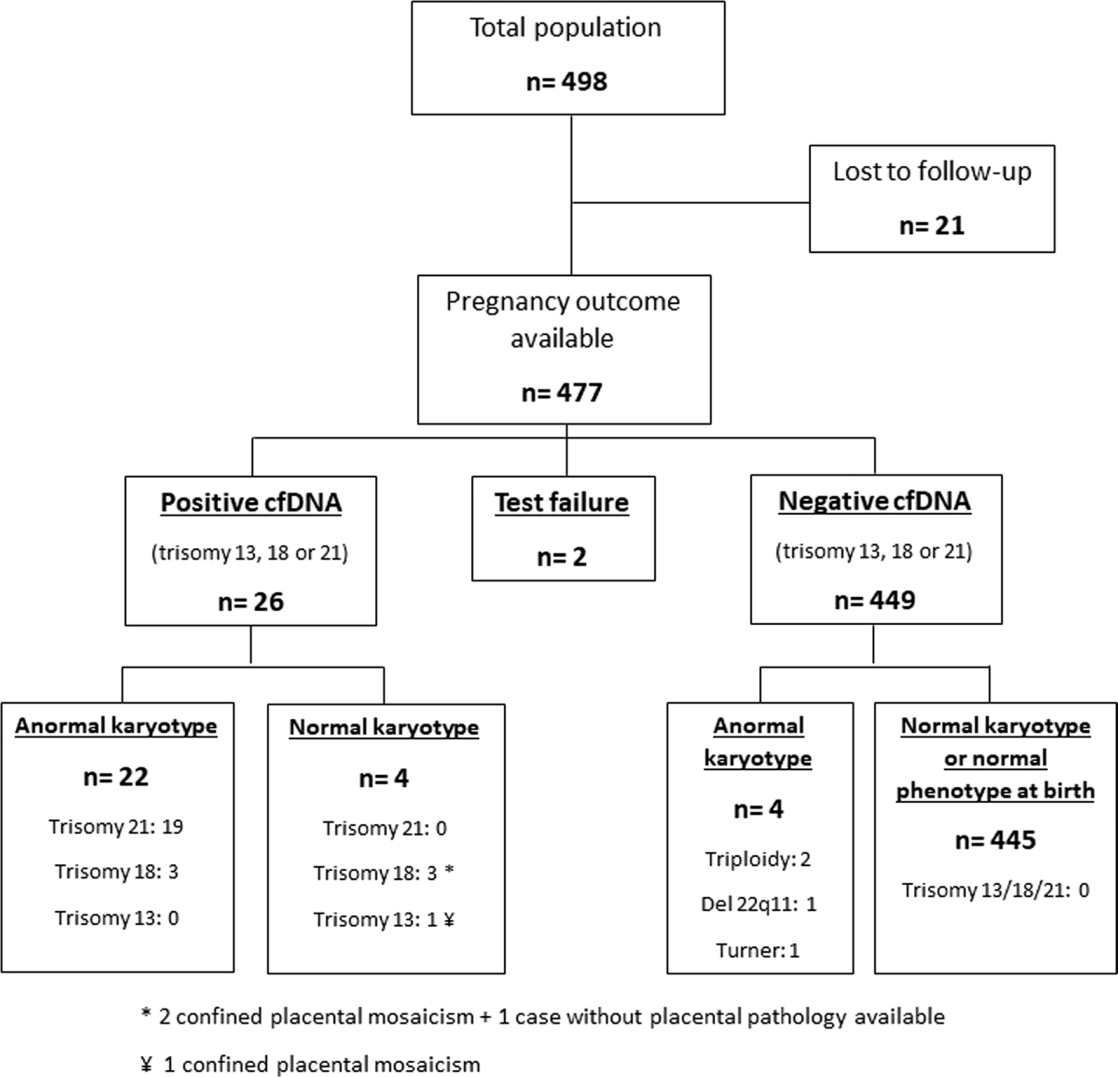
Flowchart of the study

Between June 2016 and July 2017, 498 patients with atypical first trimester serum screening profiles met inclusion criteria. We excluded 21 patients (4.1%) for whom we did not obtain pregnancy outcome. The flowchart of our study is resumed in Fig. 1. The mean maternal age was 32.8 years, the mean weight was 63 kg. The mean gestational age at blood sampling for cfDNA was 15.2 WG (range 11.4–34.1 WG). The mean fetal fraction was 11.0% (range 1.0–31.8%). Table 1 resumes the main patient characteristics. In our study cohort, 391 (82%) patients had a combined risk superior to 1/1000 after first trimester screening.

**Table 1:** Main characteristics of the study population (n = 447)

Maternal characteristics	Median (range) or n (%)
Age (years)	32.8 (19.5–46.6)
Weight (kg)	63 (40–173)
Multiparous	266 (59.5%)
Ethnicity	
Caucasian	394 (88.1%)
Other	53 (11.9%)
Amp induced pregnancy	25 (5.6%)
Auto-immune disease	24 (5.4%)
Anticoagulant treatment	9 (2.0%)
Gestational age (wg)	15.2 (11.4–34.1)
Fetal fraction (%)	11.0 (1.0–31.8)

*AMP* assisted medical procreation

### Test Performance for screening of trisomy 21, 18 and 13

Out of the 477 patients analyzed, a “no-call” result occurred for four patients initially, reduced to two patients (0.4%) after redrawn. For both patients, results were not reported because of a low fetal fraction. One of them gave birth to a phenotypically normal newborn after a normal pregnancy follow up, and the other was diagnosed with a stillbirth at 19 WG in a context of severe fetal growth retardation, with a normal karyotype and vascular abnormalities on the placenta. Among the 477 patients, 26 had a positive cfDNA assay. Twenty-two were confirmed by fetal karyotype after amniocentesis. For the four remaining patients, amniocentesis found a normal karyotype, with a normal pregnancy follow-up. A confined placental mosaicism was confirmed for three of them after birth (for the last one, no placental analysis was performed). In this group of 26 cfDNA positive tests, 25 (96.2%) of patients had a combined risk superior to 1/1000 after first trimester screening test.

Among the 449 patients with a negative cfDNA assay, 445 were confirmed by a normal karyotype performed during pregnancy for other reason or a normal phenotype at birth. No case of trisomy 21, 18 or 13 was discovered after birth. In four cases, a different chromosomal anomaly was found during pregnancy (one Turner syndrome, two triploïdy and one Di-George syndrome), screened thanks to the systematic routine ultrasound examinations performed at the second and third trimester, as recommended in France for all pregnant women. Overall, the specificity of the cfDNA assay in our study population was 100% (CI 95%, 99.2–100.0) for trisomy 21, 99.4% (CI 95% 98.2–99.9) for trisomy 18 and 99.8% (CI 95% 98.9–100.0) for trisomy 13. Specificity and sensitivity calculations are detailed in Table 2.

**Table 2:** Performance of cfDNA assay for screening of trisomies 13, 18, 21

	Sensitivity	Specificity
Trisomy 21	19/19 = 100% (CI 95% 82.4–100)	458/458 = 100% (CI 95% 99.2–100)
Trisomy 18	3/3 = 100% (CI 95% 29.2–100)	471/474 = 99.4% (CI 95% 98.2–99.9)
Trisomy 13	0/0	476/477 = 99.8% (CI 95% 98.9–100)

*CI* confidence interval

### Test performance for screening of vascular complications

For this analysis, we excluded all patients with chromosomal abnormalities (all positive cfDNA assays and the four patients with other chromosomal abnormalities). Indeed, these patients are more exposed to IUGR, as it can often be a part of the fetuses’ pathology, and not reflect a placental dysfunction. In addition to that, most patients of this group asked for termination of pregnancy, which occurred in most cases early in pregnancy according to French regulation.

Within the 447 remaining patients, four cases (0.9%) of PIH, 10 cases (2.2%) of PE and five cases (1.1%) of early PE were reported. For these events, no predictive factor was identified in the univariate or multivariate analysis. In the subgroup analysis, there was a trend of correlation between PE and the subgroup of low levels for both PAPP-A and HCG, compared to the group of both elevated levels or mixed, but it did not reach statistical significance (Fig. 3).

**Fig. 2:**
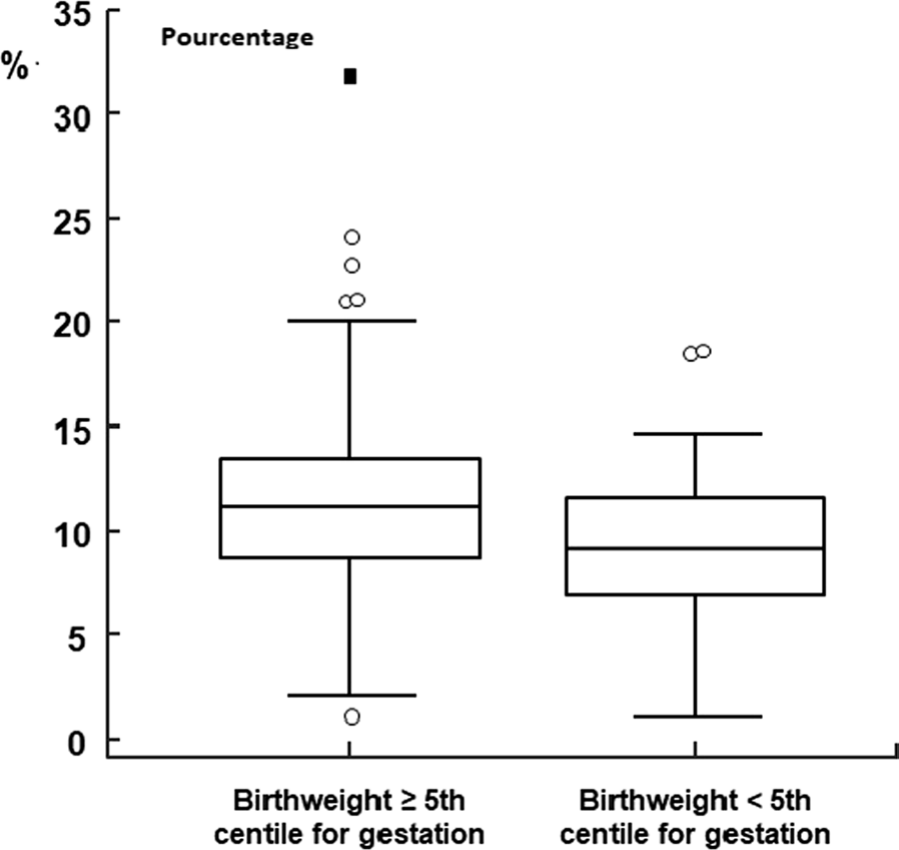
Distribution of fetal fraction on the group of small for gestational age < 5th percentile versus the rest of the study population

**Fig. 3:**
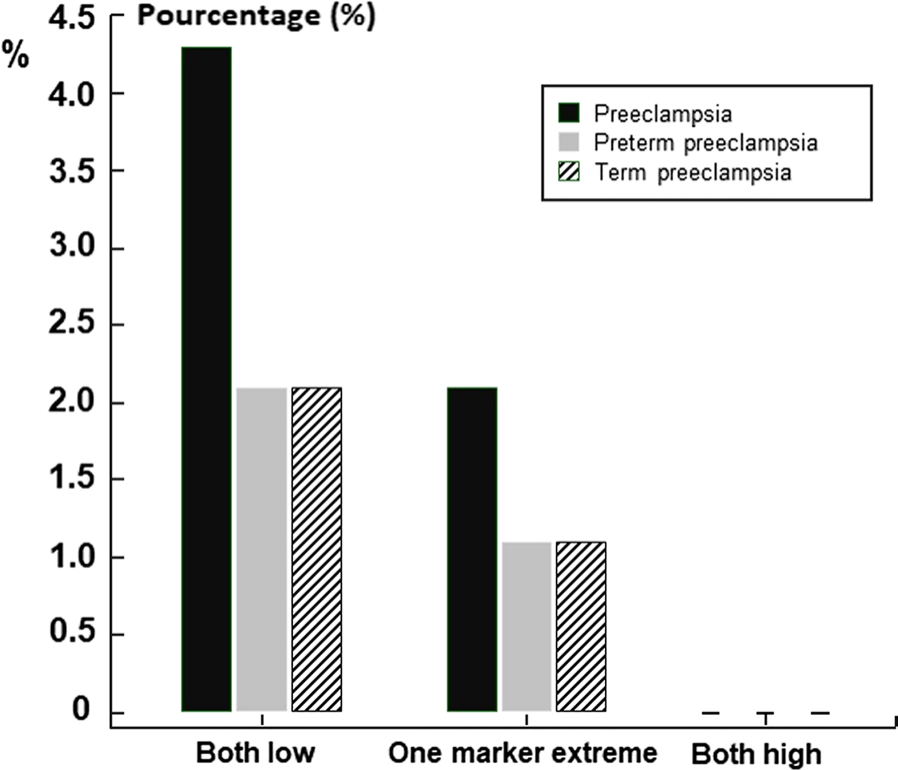
Subgroup study for correlation with preeclampsia

IUGR concerned 65 patients (14.5%), for which a statistical correlation was made with maternal age (OR 1.08, CI 95% 1.02–1.16, p = 0.015), PAPP-A level (OR 0.37, CI 95% 0.24–0.57, p < 0.0001) and medically assisted procreation (OR 4.26, CI 95% 1.54–11.75, p = 0.005). No association with fetal fraction calculated in cfDNA assay was observed (OR 0.95, CI 95% 0.87–1.02, p = 0.18). On the other hand, severe IUGR under 5th percentile (n = 44, 9.8%) were significantly associated with a low fetal fraction in cfDNA assay (OR = 0.87, ICI 95% 0.79–0.96, p = 0.006), in addition to gestational age at cfDNA assay (OR = 1.12, CI 95% 1.02–1.25, p = 0.019), PAPP-A level (OR 0.31, CI 95% 0.17–0.59, p = 0.0003) and medically assisted procreation (OR = 3.75, CI 95% 1.18–11.90, p = 0.025). These findings are detailed in Table 3, Figs. 2 and 3.

**Table 3:** Results of the logistic regression concerning IUGR < 5th percentile and maternal characteristics

Predictive factor	Odds ratios	CI 95%	p
Maternal age	1.0677	0.9896–1.1521	0.0910
Fetal fraction	0.8727	0.7908–0.9631	0.0068
Gestationnal age	1.1289	1.0202–1.2491	0.0189
HCG level	1.0822	0.9487–1.2344	0.2395
Caucasian origin	2.1622	0.5999–7.7932	0.2385
PAPP-A level	0.3127	0.1660–0.5891	0.0003
Multiparity	1.0451	0.5179–2.1088	0.9020
AMP	3.7490	1.1810–11.9007	0.0249
Maternal weight	0.9958	0.9714–1.0209	0.7417

*CI* confidence interval, *HCG* human chorionic gonadotropin, *PAPP-A* pregnancy-associated plasma protein A, *AMP* assisted medical procreation

## Discussion

### Principal findings of the study

Our study has shown that in singleton pregnancy with a normal first trimester ultrasound examination and an atypical value of first trimester serum assay i.e. HCG < 0.25 MoM, HCG > 5.0 MoM and/or PAPP-A < 0.25 MoM, PAPP-A > 2.5 MoM, cfDNA assay has a low failure rate and an excellent performance in screening for major trisomies. CfDNA can therefore be used as a screening test is this specific population. Further, we did not show a significant association between atypical biochemical markers and the occurrence of PE but an association between fetal fraction level and birthweight below the 5th percentile for gestational age.

### Comparison with results of previous studies

Patients with atypical first trimester screening serum results are at higher risk of chromosomal anomalies than general population [3]. Our aim was to explore whether cfDNA assay could be interesting in this specific population. Indeed, we found that this test has an excellent sensitivity and specificity for screening for major trisomies, and encourages us using this test for these patients. To our knowledge, this is the first study exploring the usefulness and reliability of cfDNA in patients with atypical serum results.

One of the fears around the widening use of cfDNA is to ignore other chromosomal anomalies than trisomy 21, 18 and 13, since the number of invasive procedures for fetal karyotype is reduced. In 2014, Petersen et al. reported a wide observational study based on a national database of 193,000 patients in Denmark showing that within patients with a chromosomal anomaly on karyotype, 23% of them would have been missed by cfDNA, but all of them were screened by ultrasound follow up [18]. Lindquist et al. also reported a series where the risk of missing another chromosomal anomaly was reduced by 90% when offering invasive procedure to patients with high combined risk, HCG or PAPP-A results below 0.2 MoM or any ultrasound anomaly [19]. Our results tend to reinforce the idea that the use of cfDNA does not inflate the risk of missing other chromosomal anomalies, even in this particular population, as only 4 patients were concerned, all diagnosed by ultrasound follow-up.

Due to its excellent performance rate, it has been suggested to simply remove the first trimester combined risk calculation, and directly offer cfDNA assay to all pregnant women [20]. Nevertheless, by applying such strategy, one could argue that we would miss a certain number of information concerning pregnancy risks, including vascular complications, as shown by Dukhovny et al. [21]. Indeed, it is suggested that patients with low PAPP-A level are exposed to higher risk of PE and PIH [12, 22–24], as well as IUGR [22, 23]. A low level of HCG could also be associated with a high risk of IUGR [22, 25, 26].

Although literature on the subject is rich, a large body of research have shown that the best strategy to predict and prevent occurrence of PE is based on a model taking into account maternal characteristics, physical and biochemical markers including measurement of placental growth factor rather than PAPP-A or HCG [27–32].

Several authors have explored a possible link between cfDNA assay and vascular complications, studying in particular the fetal fraction of cfDNA calculated for each patient. The main physiopathological hypothesis behind this link is that PE could cause an oxidative stress, which increases placental apoptosis [33] and cfDNA spreading. Therefore, the level of placental DNA should increase in maternal blood for these patients [11, 34–36]. It has been shown that this change in fetal fraction occurs up to 3 weeks before the first clinical symptoms of vascular complications [11]. Although this theory seems logical, clinical findings in literature are heterogeneous, as several authors find a lower rate of fetal fraction for these patients [37–39]. Other authors only find a link between fetal fraction and early PE [9, 36]. In our study, there was no significant association between fetal fraction and the occurrence of PIH or PE, due to a very low incidence of these maternal vascular events, and therefore a lack of statistical power.

Concerning fetal growth restriction, we found a significant association with a low fetal fraction. Previous authors have shown an association between low fetal fraction and IUGR < 37 weeks [38].

### Implication for clinical practice

CfDNA was initially promoted as a second-line test for pregnancies already at high risk of major trisomies based on conventional screening tests [40]. CfDNA is now considered as an acceptable first-line screening test and some countries like Belgium introduced it to all pregnant women at any background risk, yet the biochemistry test is not used any more as it cannot be cumulated for reimbursement with the use of cfDNA. There may be 2 concerns in such a strategy: first, the performance of cfDNA in patients with atypical biochemical results and second, the risk of reduction in the detection of atypical chromosome abnormalities previously identified through diagnostic testing after atypical biochemistry. Our study dismissed both of these concerns and as a consequence showed the possibility of not using any more PAPP-A and HCG in the first trimester screening for major trisomies.

### Strength and limitations

The strength of our study is that it is a multicentric study, concerning patients from all over the country, which makes our cohort representative of the general French population and leads us to think that these results can be extrapolated. Further, we managed to obtain a large percentage of pregnancy outcome, with only 4.2% of lost to follow up, thanks to a wide respond from health care practitioners all over France.

A limitation of our study is that the low incidence of PIH and PE did not allow us to obtain a sufficient statistical power to conclude on a link between vascular events and fetal fraction in cfDNA. On the other hand, our study was not primarily designed to study this association, which would have been better done if all cases with normal biochemistry results were also included in this study. Another limitation on this point is the fact that our cohort only implied patients with atypical results of HCG and/or PAPP-A, without any control group. Nevertheless, the aim of our work was not to prove the higher risk of vascular complications in this group compared to controls, which is widely established in literature, but to specify the potential contribution of cfDNA in this topic.

## Conclusion

Performance of cfDNA assaying for trisomy 21 in a cohort of patients with atypical first trimester screening serum results seem to be similar to that reported in the general population. The number of cases of trisomies 18 and 13 is at the moment too small for accurate assessment of predictive performance of the cfDNA assay in this particular population. In the coming years, we may witness a decrease in the price of cfDNA assaying, especially with the introduction of new affordable technologies that may compete with the price of the biochemical tests [41]. In many advanced countries, this may be the end of the use of PAPP-A and HCG in the first trimester screening for major trisomies.

## Data Availability

The datasets used and/or analysed during the current study are available from the corresponding author on reasonable request.

## Abbreviations

AMP: assisted medical procreation
cfDNA: cell free DNA
CI: confidence interval
HCG: human chorionic gonadotropin
IUGR: intra uterine growth restriction
MoM: multiple of median
NIPT: non invasive prenatal testing
OR: odd ratio
PAPP-A: pregnancy-associated plasma protein-A
PE: preeclampsia
PIH: pregnancy induced hypertension
WG: week of gestation
